# Pedestrians and the Built Environment during the COVID-19 Pandemic: Changing Relationships by the Pandemic Phases in Salt Lake County, Utah, U.S.A.

**DOI:** 10.1177/03611981221083606

**Published:** 2022-05-11

**Authors:** Keunhyun Park, Patrick A. Singleton, Simon Brewer, Jessica Zuban

**Affiliations:** 1 Department of Forest Resources Management, University of British Columbia, Vancouver BC, Canada; 2 Department of Civil and Environmental Engineering, Utah State University, Logan, UT; 3 Department of Geography, University of Utah, Salt Lake City, UT; 4 Department of Botany, University of Otago, Dunedin, New Zealand

**Keywords:** pedestrians, bicycles, human factors, pedestrians, planning and analysis, environmental analysis and ecology, land use planning, travel survey methods, pedestrian and bicycle data

## Abstract

The COVID-19 pandemic has dramatically altered people’s travel behavior, in particular outdoor activities, including walking. Their behavior changes may have prolonged effects after the pandemic, and such changes vary by the context and are related to the characteristics of the built environment. But empirical studies about the relationships between pedestrians and the built environment during the pandemic are lacking. This study explores how COVID-19 and related travel restrictions have affected the relationship between pedestrian traffic volume and the built environment. We estimate daily pedestrian volumes for all signalized intersections in Salt Lake County, Utah, U.S.A., from pedestrian push-button log data between January 2019 and October 2020. Multilevel spatial filtering models show that the COVID-19 pandemic has altered the relationship between pedestrian traffic volume and the built environment. During the pandemic, the higher the number of COVID-19 cases, the less (or more negative) the effects of density, street connectivity, and destination accessibility on pedestrian volume being observed. The exception is access to urban parks, as it became more significant in increasing pedestrian activities during the pandemic. The models also highlight the negative impacts of the pandemic in economically disadvantaged areas. Our findings could help urban and transportation planners find effective interventions to promote active transportation and physical activity amid the global pandemic.

An accurate prediction of pedestrian traffic volume is an essential goal for urban and transportation planners. Pedestrian traffic estimates are inputs to traffic safety analyses, health impact assessments, and guidelines on urban development. Walking activity contributes to the economic and social vitality of an urban area (*1, 2*). Because the walking activity is highly affected by the environmental context such as urban forms and streetscape, planning agencies started to model pedestrian travel demand based on built-environment data (*3, 4*).

The COVID-19 pandemic has greatly altered people’s travel behavior, in particular outdoor activities, including walking (*5–7*). The decrease in walking during the pandemic is related to fear of physical contact with other people as well as external enforced measures such as travel bans (*8*). People’s behavior changes may have prolonged effects in the new normal after the pandemic, and such changes vary by the context and are related to the characteristics of the built environment. But empirical studies about the changing relationships between pedestrians and the built environment are lacking. Without appropriate evidence, the current travel demand models may not accurately predict pedestrian traffic volume during the pandemic. A lack of (or inappropriate) interventions in the urban built environment may worsen the pandemic’s uneven impacts on different socio-economic groups.

Thus, this study explores how COVID-19 and related conditions (e.g., travel restrictions) have affected the relationship between pedestrian traffic volume and the built environment. We estimate daily pedestrian volumes for all signalized intersections in Salt Lake County, Utah, U.S.A., from pedestrian push-button log data between January 2019 and October 2020. Then, using multilevel spatial filtering models, we explain the pedestrian estimates in relation to the built-environment “D” variables—development density, land use diversity, street network design, distance to transit, and destination accessibility (Ewing and Cervero [*9*])—with the travel restriction phases as a moderator. Our results offer potential implications for travel demand modeling and forecasting, pedestrian safety analysis, and reducing the socio-economically uneven impacts of the pandemic. They could also help urban and transportation planners find effective interventions to promote active transportation and physical activity amid the global pandemic.

## Literature Review

### Impact of Pandemics on Travel Behavior

Respiratory viral pandemic outbreaks such as SARS (severe acute respiratory syndrome), H1N1, and MERS (Middle East respiratory syndrome) have negatively affected travel behavior (*8, 10–12*). Similarly, the COVID-19 pandemic has resulted in a general decrease in travel (*7, 13–17*). A study using Citymapper’s mobility index showed that mobility declined in all major cities globally throughout March 2020, and closures of public transportation, workplaces, and schools had a substantial impact on reducing population mobility (*17*).

The decrease in travel during past pandemics was found to be associated with a combination of internal motivations (e.g., perceived risks) and external enforced measures (e.g., travel bans, stay-at-home orders) (*8*). Internal motivations of perceived risk caused behavior change as people appeared to voluntarily engage in self-protection and reduce or postpone consumption to avoid risk (*8, 10*). Behavioral changes tend to happen at the beginning of the epidemic when less information is known and to lessen as time goes on (*11, 14*).

Beck and Hensher (*18*) conduct a multi-paper longitudinal travel and activity survey in Australia to understand the effects of COVID-19. Their first paper reported findings from a survey at the beginning of lockdown at the end of March. It found that 78% of respondents had already made changes to their travel behavior, the largest reduction with private cars followed by public transportation (*18*). On the other hand, respondents reported their use of active transportation increased from 14% to 20% after the COVID-19 outbreak began (*18*). The second phase of the study took place in May and June after the first outbreak leveled off and restrictions began to ease. Aggregate travel activity had increased 50% since the initial lockdown but was still less (66%) than pre-COVID-19 travel (*5*). More people reported increasing use of active transportation (e.g., walking, running, and cycling) during May and June than decreasing use (*5*). Future plans to use active modes of transportation were very similar and even more promising in the case of walking (*5*).

### Travel Preference by Mode

Other studies also show that preferences of travel mode have shifted during the COVID-19 outbreak. Perceived risk in public transportation, taxi, and ride-hailing services is higher than in private vehicles, biking, scootering, and walking (*18–20*). A study in 2009 found that the use of public transportation was a significant risk factor for contracting acute respiratory infections (*20*). In King County, Washington, USA, residents in higher-income neighborhoods chose to drive to work rather than use public transportation in the early days of the outbreak (*13*).

Social distancing measures have encouraged people to avoid areas with increased social contact and resulted in the cancellation of out-of-home activities (*21*). De Vos (*21*) hypothesizes that walking and cycling may increase as people opt for active travel to commute and for recreation. In fact, cycling has seen a surge in volume, particularly in major cities like New York and Berlin (*6*). Citi Bike, a bike-share system in New York, has had a 67% increase in ridership compared with a year ago, while subway use declined 92% (*6*). In Switzerland, bicycle use increased a significant amount, especially during weekend afternoons, suggesting an increase in leisure activity (*7*).

### Travel Reduction by Destination and the Built Environment

Decreases in travel vary by destination type. In a study using Google Mobility Reports in 771 urban counties in the U.S. (*14*), travel to parks had the least average reduction (0.4%) and the highest variation. Trips to grocery stores saw a 13.3% reduction, and trips to public transportation saw a 37.4% reduction (*14*). This may be caused in part by the higher perceived risk of infection in public transit vehicles, hospitals, restaurants, and indoor gyms and the lower perceived risk for parks and family members’ or friends’ houses (*18, 19*). On the other hand, increased risk perception at one’s workplace did not significantly reduce travel (*19*). This may be a result of economic necessity and an inability to work from home for many people (*13*).

Before COVID-19, 60% of U.S. residents in urban areas reported using online grocery shopping and food delivery from restaurants, while only 29% reported doing so in suburban areas in part because of the ease of delivery to urban areas (*14, 22*). A survey conducted in Australia during lockdown shows an 18% increase in online grocery shopping (*18*). In China, surveys show online food purchases during lockdown were most likely among young people living in large cities (*23*). People in compact developments have reduced trips to grocery stores and pharmacies (*14*), which may be caused by the observed increase in online shopping.

A study by Chang et al. (*24*) found that a small number of “superspreader” points of interest have caused a large majority of infections. For example, in Chicago, 85% of infections occurred at 10% of points of interest (*24*). These points of interest, listed in order of total cumulative infections, include full-service restaurants, religious organizations, grocery stores, limited-service restaurants, cafés and snack bars, hardware stores, automotive parts stores, the office of physicians, other general stores, fitness centers, and hotels and motels (*24*). Certain points of interest like restaurants and fitness gyms caused fewer infections than expected, most likely because of closures, and others like grocery stores caused more because they remained open (*24*).

Hamidi and Zandiatashbar (*14*) explain travel reduction by the degree of urbanization. According to their study, people who live in compact developments are more likely to have multiple shops within walking distance, resulting in a significantly higher reduction in trips to grocery stores, pharmacies, and transit stations than people in sprawling areas (*14*). The opposite holds true for park visits, where people in compact developments with smaller homes and a lack of private green spaces were less likely to reduce their trips during the shelter-in-place order (*14*).

### Travel Reduction by Socio-demographic Groups and Equity Issues

Reduction in travel varies between different socio-demographic groups during COVID-19 (*5, 14, 16, 24*). Highly educated people, older adults, and Hispanics have reduced travel more since the COVID-19 outbreak, while children and Trump voters have reduced their travel to a lesser degree than other groups (*14*). Studies on risk perception of influenza determined that males are less likely to alter travel patterns, avoid public places, and stay at home than females or people with influenza-like symptoms (*7, 15, 18*).

Lower-income households reported little change in their travel behavior during lockdown (*5, 16*). Because disadvantaged groups have not been able to reduce their travel, the places they visit (e.g., grocery stores and snack bars) are more crowded, and therefore pose a higher risk (*24*). This difference in equity is important because it represents both causes and consequences of the effects of the pandemic. Because more-disadvantaged groups travel at higher rates than more-educated and higher-income groups during the pandemic, higher viral transmission rates can occur (*13*).

Travel behavior during the COVID-19 pandemic also showed socio-demographic disparities in the use of public transportation (*5, 13*). Residents of more-educated neighborhoods were able to engage in a greater degree of mode substitution, using cars instead of public transportation (*13*). Lower-income groups have lower access to cars and are dependent on public transportation, and are relatively unable to work from home (*5, 13, 21*).

### Conceptual Framework

The COVID-19 pandemic has had a significant effect on people’s travel behavior globally, but empirical studies on pedestrian traffic volume and its relationship to the built environment are lacking. The observed effects on travel behavior are also limited by the study period. While some studies show that travel reduced during the initial months of the pandemic (*7, 13–17*), there are limited studies showing how travel trends changed over time. The normalization of the situation over time likely positively affected increased travel behavior as it did during the MERS outbreak (*11*), but there are no studies to support this. In relation to active transportation, few studies have accounted for actual use instead of relying on self-reported surveys and company reporting, which may not reflect reality (*5, 6, 18*).

**Figure 1.**
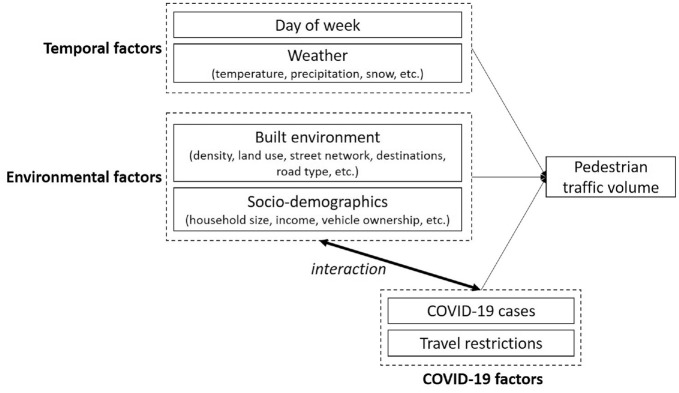
Conceptual framework of pedestrian traffic volume during the COVID-19 pandemic.

Using pedestrian volume estimate data from January 2019 to October 2020, this study explores how COVID-19 has affected the relationship between pedestrians and the built environment. Figure 1 shows the conceptual framework of this study. The COVID-19-related conditions might have significant impacts on people’s walking behaviors. Not only as an explanatory variable of the pedestrian volume, the COVID-19 factors are also associated with other variables, particularly environmental factors. As discussed above, travel reduction during the pandemic varies by destination type, built environmental characteristics, and socio-demographics of a neighborhood (*5, 13, 14, 19, 24*). While the impacts of the pandemic on the urban form changes are not dramatic in the short term (e.g., open street campaigns), pandemic-related conditions (e.g., travel restrictions, fears of infection in gathering places, employment status change) may modify how the built environment affects walking behavior. For example, people may avoid visiting a dense urban center when (actual or perceived) COVID-19 cases are high. In this study, we model such a moderating effect through interaction terms between COVID-19 factors (e.g., case counts, administrative phases) and each of the environmental variables (*25*).

In the literature, built-environment characteristics that predict pedestrian volumes, such as development density, land use diversity, street network design, destination accessibility, and distance to transit, are often called “D” variables (*9*). Neighborhood socio-demographic attributes also explain pedestrian volumes, which are higher in areas with lower-income, bigger households, and fewer cars (*26, 27*). Temporal factors explaining walking include the day of the week and weather (*28–31*).

## Data and Methods

The study area is Salt Lake County, the most populous county in Utah (a population of 1,160,437 in 2019) and home to the state capital, Salt Lake City (a population of 200,567 in 2019). Like many other U.S. regions, Salt Lake County is mostly automobile-oriented, partially a result of large blocks, wide roads, and sprawl developments.

We performed a multilevel analysis because the data involved two sets of units. Our level 1 units are days, which is a feasible unit for analyzing traffic volumes across time and space. In 2020, we included data for all days in the first 10 months (January–October). To provide a baseline against which to compare changes in built-environment relationships with walking, we also included data for all days in 2019 (the full 12 months). Thus, our analysis used up to 670 days (level 1 units). Our level 2 units are intersections with traffic signals, of which there are around 1,055 in the study area. Since some of these sites had no pedestrian push buttons (the source of our dependent variable; see next subsection), our analysis used 904 signals (level 2 units). As a result, there were a total of 520,736 observations (days × signals), which was less than theoretically possible, since some signals were missing pedestrian signal data on certain days.

### Dependent Variable: Pedestrian Traffic Volume

The dependent variable of the analysis was daily total pedestrian crossing volumes, measured at each signal on each day. The pedestrian traffic volumes were estimated from data on pedestrian push-button presses, which were recorded by high-resolution traffic signal controller event logs (*32*) and archived in a central database—the Automated Traffic Signal Performance Measures (ATSPM) system (*33, 34*) by the Utah Department of Transportation (UDOT). Recent research in Oregon (*35, 36*), Utah (*37*), and Arizona (*38*) has found pedestrian signal actuations to be strongly correlated with pedestrian crossing volumes (a correlation coefficient of 0.80 or higher), and models using push-button presses have been able to predict pedestrian crossing volumes with low average absolute error (±3 pedestrians per hour or less). These kinds of pedestrian signal data have also been used recently to examine associations with built-environment characteristics (*27*) and weather (*39*).

In this paper, we first assembled pedestrian traffic signal data from UDOT’s ATSPM system for every signal in our study area for 2019 and 2020. After processing and cleaning the data (to note missing data), we applied pedestrian volume estimation methods developed by Singleton and Runa (*37*), which we will summarize briefly. Based on manual counts of almost 175,000 pedestrians during more than 10,000 hours of video recorded at 90 randomly selected Utah signals in 2019, those authors developed non-linear (quadratic and piecewise linear) regression models predicting hourly pedestrian crossing volumes as a function of measures of pedestrian signal data. The models were of the following forms:

$$Y=\beta_{1}X+\beta_{2}X^{2}\;or\;\;\;Y=\beta_{1}X+\beta_{2}\max\left(X-{\mathrm{br}}_{1},\;0\right)$$

where Y was the observed pedestrian count, X was the pedestrian signal data measure, $\beta_{1}$ and $\beta_{2}$ were estimated model coefficients, and $br_{1}$ was the optimized piecewise linear breakpoint. The best-fitting measures of pedestrian signal data X in the regression models were unique pedestrian detections (push-button presses, removing those within 15 s of a prior press) and pedestrian actuation (the number of pedestrian phases with one or more push-button presses). The models also included some segmentation by signal type (pedestrian hybrid beacon versus traditional signal), cycle length (shorter versus longer), pedestrian push-button volume (low versus high pedestrian activity), and pedestrian operations (phase on pedestrian recall or not). Using hourly crossing data from more than 22,500 observations, the correlation between observed and model-predicted hourly pedestrian crossing volumes was 0.84, with an average prediction error of ±3.0 pedestrians per hour. (See Singleton and Runa [*37*], and Singleton et al. [*40*] for detailed information on these methods.)

Returning to the present study, after applying these pedestrian volume estimation models to our 2019 to 2020 pedestrian data at 904 signals in Salt Lake County, we then aggregated these results (over hours in a day and crossings at an intersection) to get our dependent variable: pedestrian crossing volumes for each signal and day. Although endogeneity is always a concern when using estimated data as the dependent variable in a model, we do not suspect this to be an issue here. The models used to estimate pedestrian volumes (*37*) relied almost entirely on traffic signal data. They did not include any of the environmental or temporal variables (day of week, weather, built environment, socio-demographics) that were used in this study, described in the following sections.

### Level 1 Variables

Level 1 variables are potentially defined by (and vary across) both the level 1 units (days) and the level 2 units (signals). In practice, most of our level 1 variables are measured on a daily basis, but where the same values are applied across all signals.

Pedestrian activity is affected by weather, seasonality, and climatic differences (*28–31*). Therefore, to control for these effects (which could otherwise be attributed to COVID-related changes), we included daily weather variables, including temperature (in degrees Fahrenheit), precipitation (in inches), and snowfall (true or false). Weather data were collected from the Global Historical Climatology Network (GNCH) daily, a product of the National Centers for Environmental Information (*41*). Specifically, weather data for all signals came from the weather station at Salt Lake International Airport (station USW00024127). While located at the northern edge of Salt Lake County, the airport station had complete data and is located in the same valley as the study area signals, only 23 mi from the furthest signal. In addition to the continuous temperature variable, we added a dummy variable for 90° in Fahrenheit or higher because our data suggested the non-linear effect of temperature. Average pedestrian volume dropped significantly during days of 90°F or higher. Another approach to handle such a non-linear effect can be adding a squared temperature variable. The COVID-19 phase variables were also defined using the same dates for all signals in the study area. Dates were taken based on state and county guidelines that mandated or recommended certain restrictions on businesses, organizations, gatherings, and travel. These are intended to measure the effects of government policies and travel restrictions as well as the public’s perceptions of and reactions to rising COVID-19 case counts, test positivity rates, hospitalizations, death counts, and so forth. The four phases we defined in this study are:

- Pre-COVID (Baseline; January 1, 2019–March 5, 2020).
- Phase 1 (High risk; March 6, 2020–April 30, 2020): On March 6, 2020, the Utah governor declares a state of emergency. Then, Utah K-12 schools take a 2 week pause from in-person classes. Utah colleges and universities cancel in-person classes for the remainder of the semester. The Church of Jesus Christ of Latter-day Saints (with which the majority of Utahns affiliate) suspends in-person gatherings. Between March 27 and 29, the Utah governor, Salt Lake County mayor, and Salt Lake City mayor issue stay-at-home directives, orders, and proclamations, with exceptions for essential activities and work. On April 17, 2020, the Utah governor introduces a color-coded system, in which the state is at a “high-risk” (red) level. Salt Lake County issues an order encouraging face coverings in public places.
- Phase 2 (Moderate risk; May 1, 2020–October 12, 2020): On May 1, 2020, the Utah governor moves the state to a “moderate-risk” (orange) level. Salt Lake County order gradually allows businesses to reopen with social distancing requirements. About 2 weeks later (May 16), the Utah governor moves the state to a “low-risk” (yellow) level, but Salt Lake City and West Valley City (in Salt Lake County) remain at a “moderate-risk” (orange) level.
- Phase 3 (Second peak; October 13, 2020–October 31, 2020): With increases of new COVID-19 cases, hospitalizations, and deaths, the Utah governor updates statewide guidelines with a new county-based system depending on new cases, test positivity rates, and statewide hospitalizations on October 13, 2020. Salt Lake County is rated as having “high transmission,” and face coverings are required in indoor public places and outdoors where social distancing is not possible.

The final level 1 variable is the “pedestrian recall” dummy variable, which (unlike the other level 1 variables) is true only for certain days and certain signals. To reduce the spread of COVID-19 through touching infected surfaces, several dozen traffic signals in and around downtown Salt Lake City were placed on pedestrian recall on April 17, 2020. Signs were placed instructing pedestrians to not push the pedestrian push-button, and traffic signals automatically brought up the walk signal every cycle. On June 29, 2020, the signs were removed, and the traffic signals went back to normal actuated operations, in which pedestrians need to press the button to receive the walk signal (most of the time) (*42*). The signals and days when the full pedestrian recall was in effect have been included in the model. For a sensitivity test, we ran models without those signal-days of pedestrian recall. Neither the signs nor the statistical significance of all coefficients of the two models (see below about the model details) changed, except for that in the phases model, the *p*-value for the Phase 1 variable increased from .020 to .054 (i.e., marginally significant).

### Level 2 Variables

Level 2 variables are only defined (and vary across) the level 2 units (signals). These variables include measures of the built environment, transportation system, and neighborhood surrounding each signalized intersection. Information about population and employment density, residential and commercial land uses, four-way intersections, public transit stops, schools, places of worship, and parks were assembled using ¼-mile (400-meter; 5-minute walking distance) network buffers around each signal. Additionally, neighborhood socio-demographic characteristics like median household income, average household size, and average vehicle ownership were calculated. Such data were obtained from the American Community Survey (ACS) 2013 to 2017 (Census block groups), the Longitudinal Employer-Household Dynamics (LEHD) program (Census blocks), the Utah Automated Geographic Reference Center (AGRC) for 2019 (parcels and places), and OpenMobilityData for 2019 (transit stops). We checked the multicollinearity among the explanatory variables, and none of those had a variance inflation factor (VIF) of 10 or higher. See Table 1 for definitions and descriptive statistics of independent and dependent variables.

**Table 1.** Definitions and Descriptive Statistics of Independent and Dependent Variables

Variable	Definition	Mean (proportion for dummy variables)	Standard deviation
LEVEL 1: day (*n* = 520,736; 670 days per intersection)			
Pedestrian traffic volume	Estimated daily total pedestrian crossing volumes, measured at each signal on each day from January 1, 2019 to October 30, 2020 (see 37 for methodology)	353.90	39,236.82
Daily COVID-19 case counts	Daily COVID-19 case counts for Salt Lake County	80.54	151.27
Weekend (dummy)	1 = yes, 0 = no	0.29	0.45
Daily temperature	In degrees Fahrenheit	58.12	18.85
Daily temperature (90° or higher; dummy)	1 = yes (90° Fahrenheit or higher), 0 = no	0.003	0.06
Daily precipitation	In inches	0.04	0.12
Snow (dummy)	1 = yes, 0 = no	0.07	0.26
Pedestrian recall (dummy)	1 = yes (pedestrian recall between April 20th and June 29, 2020 in and around downtown Salt Lake City), 0 = no	0.01	0.08
LEVEL 2: intersection and ¼-mile buffer (*n* = 904)			
Population density	1,000 population per square mile (2.59 km^2^)	4.68	2.55
Employment density	1,000 jobs per square mile (2.59 km^2^)	7.43	12.69
% Residential parcels	Percentage of residential parcels	36.51	25.10
% Commercial parcels	Percentage of commercial parcels	29.42	21.79
% 4-Way intersections	Percentage of 4-way intersections	25.46	19.00
Public transit stops	Number of public transit stops	5.65	4.16
Schools	Number of K-12 schools	0.32	0.63
Places of worship	Number of places of worship	0.53	0.86
Park (acre)	Total acreage of parks	1.84	4.35
Median household income	Median household income ($1,000)	63.03	24.24
Household size	Average number of people per household (log-transformed)	2.90	0.94
Average vehicles	Average number of cars per housing unit	1.60	0.51
Major road (dummy)	1 = yes (signals located within 30 m from major arterials), 0 = no	0.58	0.49
Salt Lake City (dummy)	1 = yes (signals located within Salt Lake City), 0 = no	0.36	0.48

### Methods

The structured nature of these data suggested that a multilevel modeling approach was appropriate, with daily counts of pedestrian activity nested within intersections. While multilevel models address within-intersection correlation of pedestrian counts, they do not account for spatial structure in the data, that is, the similarity in counts for intersections that are located close together. The basic multilevel model described above was tested for residual autocorrelation using Moran’s *I*. The results (*I* = 0.09, *Z* = 3.39; *p* = 0.00035) indicated significant spatial autocorrelation and therefore lack of independence in the errors. Spatial autocorrelation can be accounted for in multilevel models using covariance functions (*43*). However, this requires the estimation of two different random effects, which can be computationally demanding for large data sets. Griffith (*44*) proposed an eigenvector spatial filtered multilevel (ESF-ML) model approach as an alternative approach. In spatial filtering, residuals from a model are decomposed into a spatially varying error and a random noise term (*45*). A spatial filter is then created by iteratively selecting spatial patterns that, when aggregated together, match the spatially varying error. The filter is subsequently incorporated as an extra term in the model. The filter “whitens” the model residuals by removing a spatial dependency. While spatial filtering has been widely applied to non-multilevel models (*46*), applications to multilevel models are rarer. However, this method has been successfully applied to a variety of outcomes, including migration flows (*47*), health outcomes (Park and Kim [*48*]), and house prices (*49*).

For a spatio-temporal data set, the ESF-ML model is written as

$$y_{i,t}=\beta X_{t}+\gamma Z_{i}+\delta E_{i}+b_{i}+\epsilon_{i,t}$$

where $y_{i,t}$ is the pedestrian count for intersection i at time t ; $X_{t}$ is the set of time-varying covariates at time t (see “LEVEL 1” variables in Table 1); $Z_{i}$ is the set of intersection-level covariates (see “LEVEL 2” variables in Table 1); $b_{i}$ is the random effects for intersection i ; and $\epsilon_{i,t}$ is random unstructured noise. Both $b_{i}$ and $\epsilon_{i,t}$ are assumed to be normally distributed with a mean of zero. $E_{i}$ is the set of spatial eigenvectors (EVs) for intersection i . β , γ, and δ represent vectors of coefficients to be estimated. EVs are derived from eigen decomposition of the following matrix (Griffith [*44*]):

$$\left(I-1\;\cdot \;1^{T}/n)\;\cdot \;W\;\cdot \;(I-1\;\cdot \;1^{T}/n\right)$$

For n locations, W is a spatial weight matrix of size n×n , where non-zero entries indicate spatial connectivity between two locations. T is the transpose operator. Connectivity between locations was determined using a Gabriel graph network (*50*).

This resulted in n candidate EVs, each representing a possible spatial pattern from highly positively autocorrelated to highly negatively autocorrelated. As the EVs were created through eigendecomposition, the EVs were orthogonal and uncorrelated with each other. An iterative, stepwise procedure was then used to build the spatial filter. In the first iteration, we estimated a set of multilevel models, where each one included one EV and the set of all other covariates. Moran’s *I* was calculated for each of these models, and we selected the one that resulted in the greatest reduction in the value of *I*. The EV from that model was then retained, and the next iteration estimated a new set of models using the remaining EVs. This stepwise procedure was repeated until the autocorrelation was no longer significant (i.e., p>0.05 ). Tests with higher thresholds (e.g., p>0.1 ) resulted in no change in the direction of inference on the coefficients. The selected EVs were then used to construct the final, spatially filtered model. For the data set used here, the final filter consisted of six EVs for both models, with a Moran’s *I* value of 0.046 ( p=.050 ) for the count model, and a Moran’s *I* value of 0.045 ( p=.055 ) for the phases model, respectively. Thus, these results indicate no significant spatial autocorrelation issue. All models were estimated using restricted maximum likelihood with the lme4 package (*51*) in R 4.1.1. Code to run the spatial filter is available through https://github.com/simonbrewer/covid_signal.

## Results

### Pedestrian Traffic Volume Trend during the COVID-19 Pandemic

Overall, pedestrian traffic volume in Salt Lake County decreased 26.3% during the COVID-19 pandemic. From March through October 2019, the daily pedestrian estimate was 133 people per intersection. During the same months of 2020, the number decreased to 98. By month in 2020, the average pedestrian estimate was lowest in April (86.7 per day per intersection) and highest in September (106.3 per day per intersection).

**Figure 2.**
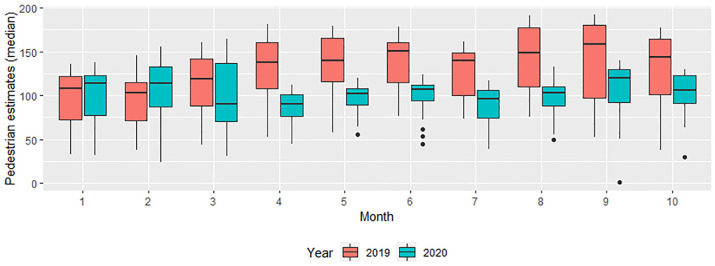
Daily pedestrian volume estimates per intersection by month in 2019 and 2020.

The pedestrian volume reduction between the two years was highest in April (53% decrease; −45.6/day) and August (44% decrease; −43.7/day) and lowest in January (1% increase; +1.4/day) and February (9% increase; +9.6/day). *T*-test results show that the differences between the two years are statistically significant in April through October, but not in January through March, at *p* < .05 significance level (Figure 2).

**Figure 3.**
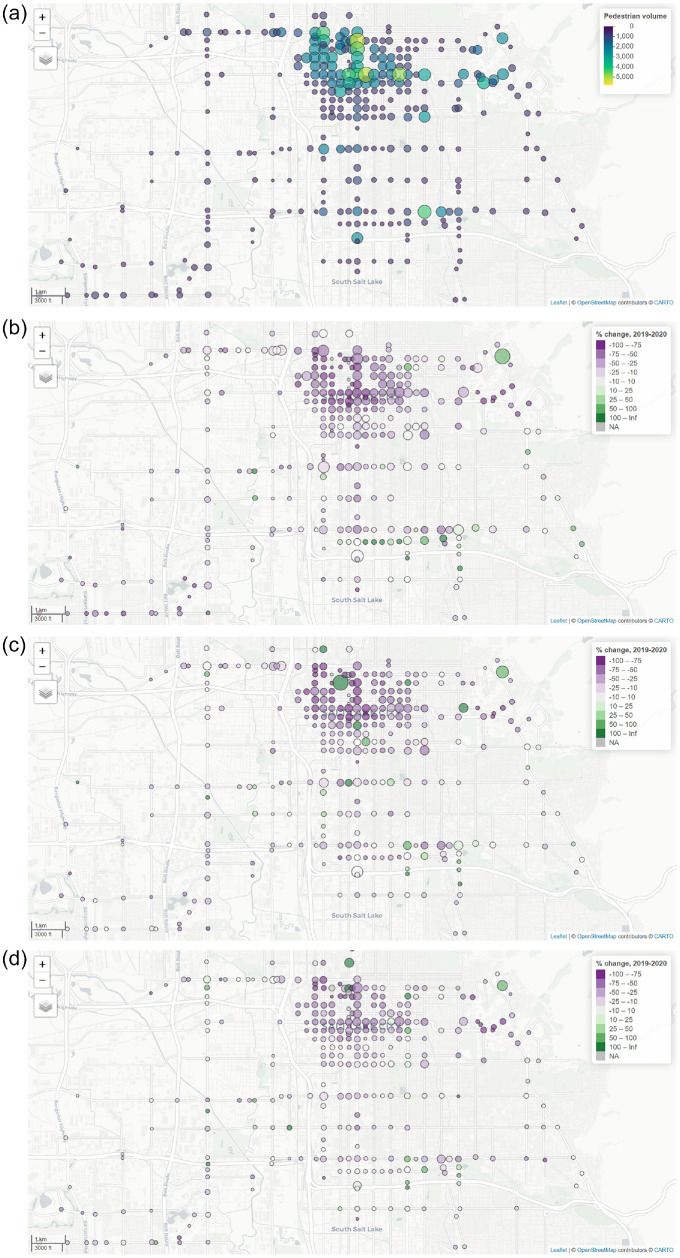
Average daily estimates of pedestrian volumes, pre-COVID and during each phase: (*a*) pedestrian volumes, pre-COVID (week centered on January 4, 2019), (*b*) pedestrian volumes, Phase 1 (high risk) (week centered on January 4, 2020), (*c*) pedestrian volumes, Phase 2 (moderate risk) (week centered on July 1, 2020), and (*d*) pedestrian volumes, Phase 3 (second peak) (week centered on October 15, 2020).

To help visualize these results, in Figure 3, we present example maps showing pedestrian volumes pre-COVID and during each of the three phases (1: high risk, 2: moderate risk, 3: second peak). To account for day-of-week variations, daily volumes are averaged over a 7 day period centered on the date specified in the figure. Each map shows average daily pedestrian crossing volumes, depicted proportionally to the area of the circles. For the COVID-19 phase maps in 2020, the color of the circle is related to the percentage change over the same week in 2019.

### Multilevel Spatial Filtering Model with Interaction Terms

Table 2 shows the results of a multilevel spatial filtering model with interaction terms with daily COVID-19 case counts. Before the pandemic, pedestrian estimates were positively related to population density, percentage of commercial land uses, public transit access, school and park access, and being in Salt Lake City, while negatively associated with a median household income of neighborhoods near the interaction.

**Table 2.** Coefficients of Variables in a Multilevel Spatial Filtering Model with Interaction Terms with Daily COVID-19 Case Counts

	Pre-COVID (January 1, 2019–March 5, 2020)	Interaction terms with daily COVID-19 cases (March 6, 2020–October 31, 2020)
LEVEL 1: day (*n* = 520,736; 670 days per intersection)		
Intercept	3.697**	
Daily COVID-19 case counts	−0.0004**	
Weekend (dummy)	−0.586**	
Daily temperature	0.004**	
Daily temperature (90° or higher; dummy)	−0.196**	
Precipitation	−0.388**	
Snow (dummy)	−0.257**	
Pedestrian recall (dummy)	−0.756**	
LEVEL 2: intersection and ¼-mi buffer (*n* = 904)		
Population density^a^	0.485**	−0.00013**
Employment density^a^	0.048	−0.00009**
% Commercial parcels	0.014**	−0.00001**
% 4-Way intersections	−0.003	−0.00001**
Public transit stops	0.065**	−0.00001**
Schools	0.135*	−0.00010**
Places of worship	0.019	−0.00021**
Park (acre)^a^	0.041**	0.000004***
Median household income	−0.007**	0.00001**
Household size^a^	−0.115	0.00011**
Major road (dummy)	0.080	0.00016**
Salt Lake City (dummy)	0.260*	−0.00006**
Random effects	Level 1 (day) variance: 1.179	
	Level 2 (intersection) variance: 0.592	

*Note*: The dependent variable is log-transformed pedestrian traffic volume estimates. Coefficients of Moran eigenvectors are hidden.

a Log-transformed.

* *p *< .05. **p < .01. ***p < .1.

The interaction terms between the COVID-19 case counts and built environmental variables were mostly negative, meaning that the higher the number of COVID-19 cases, the less (or more negative) the association of the density, street connectivity, and destination accessibility with pedestrian volume was. For example, while a 10% increase in population density near an intersection yielded a 4.85% increase in pedestrian volume pre-COVID-19, the elasticity value dropped to 4.21% when Salt Lake County had 500 daily COVID-19 cases—(0.485−0.00013×500)×10. Such a reduction was more remarkable in employment centers (elasticity of 0.048 for pre-COVID dropping to 0.004 for 500 daily COVID-19 cases) than in population centers, as hypothesized.

Likewise, we found a negative interaction term between the COVID-19 cases and the percentage of commercial parcels (an elasticity of 0.014 dropping to 0.009 for 500 cases). Among the other built-environment variables, we also observed declining associations of the percentage of four-way intersections (a measure of street network connectivity) and the number of public transit stops, schools, and places of worship with pedestrian volume as COVID-19 case counts increased. More noticeable drops were observed for access to schools (an elasticity of 0.135 dropping to 0.084 for 500 cases) and to places of worship (an elasticity of 0.019 turning negative, −0.088, for 500 cases).

Interestingly, accessible park acreage near an intersection became even more significant in increased pedestrian traffic volume during the pandemic. Unlike the pre-COVID period when pedestrians were more observed in lower-income areas, the elasticity of income became smaller as the COVID-19 case count increased. The coefficient of median household income variable ($1,000) dropped by half from −0.007 pre-COVID to −0.003 during COVID-19 with 500 daily cases. The interaction terms with COVID-19 case counts were positive with household size and a major road dummy and negative with being in Salt Lake City, which means that the normally higher level of pedestrian volume in the City was less evident during the pandemic.

In respect of the temporal variables, pedestrian traffic volume increased with daily temperature and decreased with it being a hot day (over 90° in Fahrenheit), precipitation, snow, and weekends during the past 2 years (January 2019 to October 2020). The use of pedestrian recall to avoid push-button use during COVID-19 lowered pedestrian estimates at those intersections, as expected.

Then, Table 3 shows the results of a multilevel spatial filtering model with interaction terms with three COVID-19 restriction phases. In Salt Lake County, pedestrian traffic volumes were actually higher in Phase 1 (high risk; March 6, 2020–April 30, 2020) compared with the pre-COVID period, when other temporal and environmental variables are controlled, and dropped significantly afterward. The phases coincide with different seasons. For instance, the negative interaction between schools and Phase 2 could be a “summer break” effect.

**Table 3.** Coefficients of Variables in a Multilevel Spatial Filtering Model with Interaction Terms with Three COVID-19 Restriction Phases

	Pre-COVID (January 1, 2019–March 5, 2020)	Interaction with phases		
		Phase 1 (high risk) March 6–April 30, 2020	Phase 2 (moderate risk) May 1–October 12, 2020	Phase 3 (second peak) October 13–31, 2020
LEVEL 1: day (*n* = 520,736; 670 days per intersection)				
Intercept	3.596**			
Phase 1 (high risk)	na	0.052*	na	na
Phase 2 (moderate risk)	na	na	−0.165**	na
Phase 3 (second peak)	na	na	na	−0.079*
Weekend (dummy)	–0.580**			
Daily temperature	0.006**			
Daily temperature (90° or higher; dummy)	–0.208**			
Precipitation	–0.432**			
Snow (dummy)	–0.264**			
Pedestrian recall (dummy)	–0.254**			
LEVEL 2: intersection and ¼-mi buffer (*n* = 904)				
Population density^a^	0.485**	−0.014*	−0.030**	−0.080**
Employment density^a^	0.067***	−0.081**	−0.069**	−0.018*
% Commercial parcels	0.015**	−0.006**	−0.004**	−0.004**
% 4-Way intersections	−0.002	−0.005**	−0.004**	−0.001**
Public transit stops	0.068**	−0.016**	−0.010**	−0.002
Schools	0.136*	0.005	−0.033**	−0.007
Places of worship	0.029	−0.036**	−0.082**	−0.072**
Park (acre)^a^	0.041**	−0.007**	−0.001	−0.002
Median household income	−0.008**	0.004**	0.004**	0.001*
Household size^a^	−0.093	−0.094**	−0.025**	0.127**
Major road (dummy)	0.066	0.053**	0.082**	0.023
Salt Lake City (dummy)	0.271**	0.001	−0.064**	−0.011
Random effects	Level 1 (day) variance: 1.175			
	Level 2 (intersection) variance: 0.560			

*Note*: na = not applicable. The dependent variable is log-transformed pedestrian traffic volume estimates. Coefficients of Moran eigenvectors are hidden.

a Log-transformed.

* *p *< .05. **p < .01. ***p < .1.

The results show varying relationships between the environmental variables and pedestrian estimates by the COVID-19-related restriction phases. The COVID-19 pandemic reduced the association between population density and pedestrian volume more profoundly in later phases. Aligned with the count-based model, reductions in pedestrian traffic volume were greater in areas with higher employment density and commercial land uses than those with residential density. The negative interaction terms with other D variables, including percentage of four-way intersections and the number of transit stops, were more apparent in earlier pandemic phases.

In respect of the role of urban park accessibility, pedestrian traffic volume was only negatively related to park access when the COVID-19 outbreak started (Phase 1) and then became insignificant in subsequent phases. In other words, the positive effect of park availability on pedestrian volume (an elasticity of 0.041) remains consistent.

## Discussion and Conclusions

In this study, we compare pedestrian traffic volume between 2019 and 2020 and find a 26% decrease on average, with higher reductions in April, May, and August. The pedestrian volume reductions in 2020 compared with 2019 are statistically significant in April through October but not for January through March. Then we run two spatial filtering models about the estimated pedestrian traffic volume and spatial and temporal attributes. We find that the COVID-19 pandemic and related travel restrictions have altered the relationship between pedestrian traffic volume and the pre-existing built-environment conditions, confirming our conceptual framework (Figure 1). Two spatio-temporal regression models show the statistical significance of both main effects of the COVID-19 factors and their interaction terms with the built-environment variables.

Before the pandemic, pedestrian estimates were positively related to population density, commercial land uses, access to public transit stops, schools, and parks, while negatively associated with a median household income of neighborhoods near the interaction. These results align with the impact of built-environment “D variables” on pedestrian volumes and walking, as documented in the literature (*2, 26, 29, 52, 53*). Different from the previous studies (Miranda-Moreno and Fernandes [*29*]), employment density is not a significant predictor of pedestrian volume in our models, which could be because of its correlation with the percentage of commercial parcels (*r* = 0.48; *p* < .01).

During the pandemic, however, the higher the number of COVID-19 cases, the less (or more negative) the associations between pedestrian volume and the density, street connectivity, and destination accessibility being observed (Table 2). Employment centers and places with more commercial land uses seem to have been more greatly affected than more residential areas, reflecting the impact that the pandemic has had on increasing telework and reducing public transportation use (*18, 19*). Some employment centers may not be functioning as employment centers during the lockdown. But such reductions in pedestrian activity in commercial centers may not entirely persist once the pandemic conditions recede. Compared with earlier phases, during the third phase of COVID conditions in Utah (October 13–October 31, 2020), the reduced impact of employment density has diminished (Table 3).

Our models also show moderating effects of the pandemic conditions in respect of other built-environment characteristics. At later pandemic stages, the relationships between pedestrian activities and access to schools and transit stops have returned to their pre-COVID level (Table 3). On the other hand, the declining association of access to places of worship with pedestrian volume has been consistently observed through all three stages, which might imply ongoing impacts post-pandemic.

An exception to this overall trend of reduced built-environment associations with pedestrian activity during COVID is access to urban parks. Accessible park acreage near an intersection became even more significant in increased pedestrian traffic volume during the pandemic (Table 2). Other studies also show that park visitation has increased since the COVID-19 outbreak in most countries (*54*) or decreased least compared with other types of travel during the early phases of the pandemic (*14*). People used parks and trails as a refuge to relax, exercise, and socialize, and thus, to overcome physical and mental issues caused by shelter-in-place and limited social relationships (*55*). Although this result may revert once conditions are “back to normal” and people spend more time in commercial/office/retail areas, it is possible that people have caught the habit of walking for recreation and will continue to make increased pedestrian trips near parks, as shown in Salon et al. (*56*). For all the damage it has caused, the pandemic could serve as a shock that results in sustained active and sustainable travel behavior change, as long as efforts are made to encourage continued walking activity. This trend should continue to be monitored, and investments could be made to provide easier nonmotorized access to parks, trails, and other recreational areas.

Our findings of varying influences of the built environment on pedestrian traffic volume could be used to adjust travel demand models in regional transportation planning practice. Recently, regional travel demand models started to incorporate pedestrian travel into the modeling framework thanks to the advancement of data collection (*3, 4*). Without appropriate evidence, the current travel demand models may not accurately predict pedestrian traffic volume during the pandemic or similar disruptions in travel behavior. Even if it may be unrealistic to expect regional models to become sensitive to unanticipated population-wide shocks to travel behaviors, shocks such as the COVID-19 pandemic, the findings about changes in built-environment relationships with walking suggest that travel forecasting systems should continue to become more flexible and responsive. Actions such as utilizing more up-to-date big data sources such as traffic signal data (used in this research) or passive smartphone data (*57*), and further developing the capabilities of citywide digital twins (*58*), could help transportation planning to become more robust to future disturbances.

The changes in how walking is related to density, street connectivity, and destination accessibility (especially employment centers and commercial land uses) also have the potential to affect other transportation tasks that rely on assumptions or estimates of pedestrian activity. For example, safety analysis requires measures of pedestrian exposure at the level of intersections and street segments. If the relationships inherent to direct-demand built-environment models of pedestrian volumes (*59, 60*) are changing, then static estimates of activity may not reflect true pedestrian risk. This is particularly important because preliminary 2020 U.S. traffic safety data show a troubling continued increase in pedestrian deaths despite significant decreases in vehicle distances traveled (*61*). The models also highlight the potential negative impacts of the pandemic in economically disadvantaged areas. Unlike the pre-COVID-19 period, when pedestrians were more observed in lower-income areas, the elasticity of income became smaller as the COVID-19 case count increased (Tables 2 and 3). This may imply that travel patterns (in particular, recreational walking) of economically disadvantaged people might have been more affected by the pandemic-related conditions (e.g., health, employment, relationships). For example, Morse et al. (*62*) show that higher-income individuals increased outdoor social and recreational activities during the COVID-19 pandemic more than low-income people.

These findings call for appropriate and timely policy actions to promote active transportation and physical activity among the socio-economically disadvantaged population. Promoting active travel encourages people to maintain a higher level of well-being (*21, 63*). Major cities worldwide (e.g., Berlin, Vienna, Philadelphia, Vancouver, Bogotá, and Mexico City) have either temporarily or permanently turned car lanes into sidewalks and bike lanes (*63*). Other examples to encourage active transportation during the pandemic include restricting cars from certain streets, adding cycling parking, allowing bicycles on footpaths, and reducing waiting time at crossroads for pedestrians (*18*). But Schmidt’s (*64*) study finds that such street closures were implemented more in wealthier regions, which is worse in larger cities. Thus, appropriate interventions through an environmental justice lens—for example, prioritizing open streets projects in lower-income communities, especially to access parks and public transit—are warranted to address the pandemic’s uneven impacts on different socioeconomic groups (*13, 24*).

This study has strengths as land use-transportation research in its use of big data (670 days times 904 intersections) in a city, a robust statistical approach addressing both spatial and temporal dependency, and an examination of multiple travel restriction phases and a focus on pedestrian transportation during the COVID-19 pandemic. On the other hand, a future study could expand the study sites to multiple cities or countries to ensure the external validity of the models. For example, since Salt Lake County is an urban county at the center of a larger region, different relationships and changes may be seen in smaller or more rural communities. Also, as not all the built-environment variables are easily obtainable for regional planning agencies, such as metropolitan planning organizations, more parsimonious models of predicting pedestrian traffic volume would be needed for their practical uses. Additionally, future work should continue this line of analysis to cover more recent stages of the pandemic (including when vaccines were widely available) and beyond to determine whether changes in built-environment relationships with walking persist into the new normal period. Nevertheless, this study provides a foundation for subsequent practice steps and sheds light on the importance of appropriate and timely interventions to promote active transportation and physical activity amid the global pandemic.
